# Swallowing‐induced atrial tachycardia associated with sympathetic activation: A case report

**DOI:** 10.1111/anec.12757

**Published:** 2020-04-26

**Authors:** Xinlu Wang, Junmeng Zhang, Liting Cheng, Ziyu Wang, Zefeng Wang, Yongquan Wu

**Affiliations:** ^1^ Department of Cardiology Beijing Anzhen Hospital Capital Medical University Beijing China; ^2^ Beijing Institute of Heart, Lung and Blood Vessel Diseases Beijing China

**Keywords:** catheter ablation, swallowing‐induced atrial tachycardia, sympathetic nerve activity

## Abstract

A 51‐year‐old woman presented with a 5‐year history of a bypass tract of a left posterior septal ablation for atrioventricular reentrant tachycardia (AVRT). Following the procedure, while swallowing even without any water or food, she felt a new onset of palpitations, and swallowing‐induced atrial tachycardia was diagnosed. We report on this patient with tachycardia induced by swallowing who received a comprehensive assessment. The swallowing‐induced atrial tachycardia deriving from the right pulmonary vein was cured by catheter ablation. In our case, the swallowing‐induced atrial tachycardia was connected with the activation of the sympathetic nervous system, which differs from typical reports of a vagal nerve reflex association.

## 1 INTRODUCTION

Swallowing‐induced atrial tachyarrhythmia (SIAT) is infrequent, and approximately 50 cases of SIAT have been reported in the literature. Most of cases of SIAT occur in adult men and recur frequently a short time while swallowing (Tandeter & Katz, 2010). The pathophysiology of SIAT has not been completely resolved, although several mechanisms have been suggested (Mathew, Green, & Nery, 2018).

## 2 CASE REPORT

**FIGURE 1:**
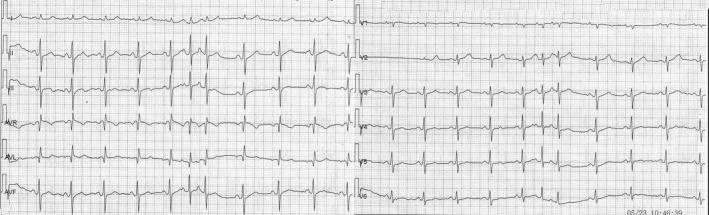
A seizure from swallowing‐induced atrial tachycardia was recorded by Holter ECG, with each episode lasting a few seconds

A 51‐year‐old woman had a 5‐year history of a bypass tract of a left posterior septal ablation for atrioventricular reentrant tachycardia (AVRT). After the radiofrequency ablation, she experienced a different kind of palpitations, which occur when swallowing with or without food or drink or when lying on her left lateral side. A Holter monitor showed premature atrial contractions (PACs) and atrial tachycardia (AT) during swallowing (Figure 1). She received metoprolol treatment, and the symptoms seemed to improve. Recently, the palpitations were worse after she ate something spicy, and propafenone did not help.

The patient did not experience symptoms of chest pain, tightness or fainting, and she had not experienced nausea or heartburn, gastrointestinal distress, difficulty with or painful swallowing. The physical examination and laboratory tests were normal. What's more, no evidences of left ventricular dysfunction or structural heart diseases were found by echocardiography.

**FIGURE 2:**
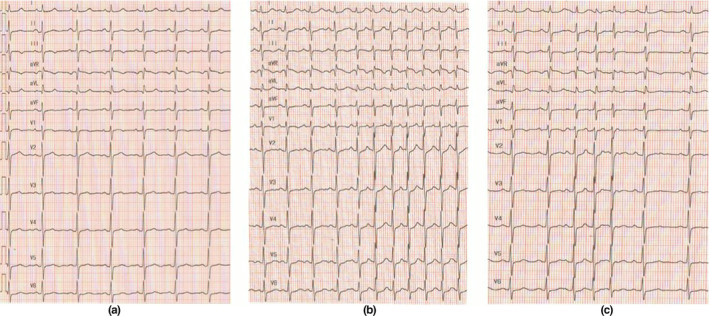
(a) A 12‐lead electrocardiogram (ECG) recording of sinus rhythm before intravenous atropine; (b) A 12‐lead electrocardiogram of paroxysmal atrial tachycardia induced by swallowing detected 5 min after intravenous atropine administration; (c) A 12‐lead electrocardiogram of paroxysmal atrial tachycardia induced by swallowing detected 20 min after intravenous atropine administration

**FIGURE 3:**
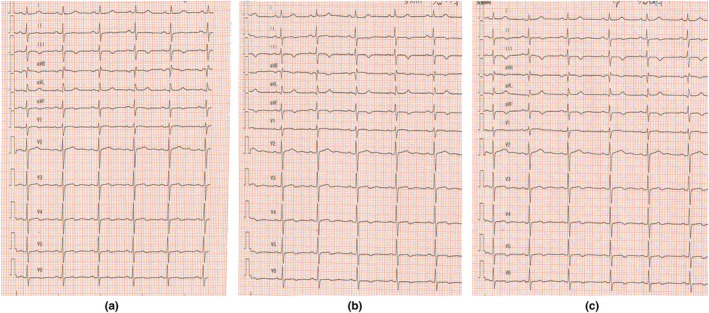
(a) A 12‐lead electrocardiogram (ECG) recording of a sinus rhythm before intravenous esmolol administration; (b) A 12‐lead electrocardiogram detected when the patient swallowed 5 min after intravenous esmolol administration; (c) A 12‐lead electrocardiogram detected when the patient swallowed 20 min after intravenous esmolol administration

**FIGURE 4:**
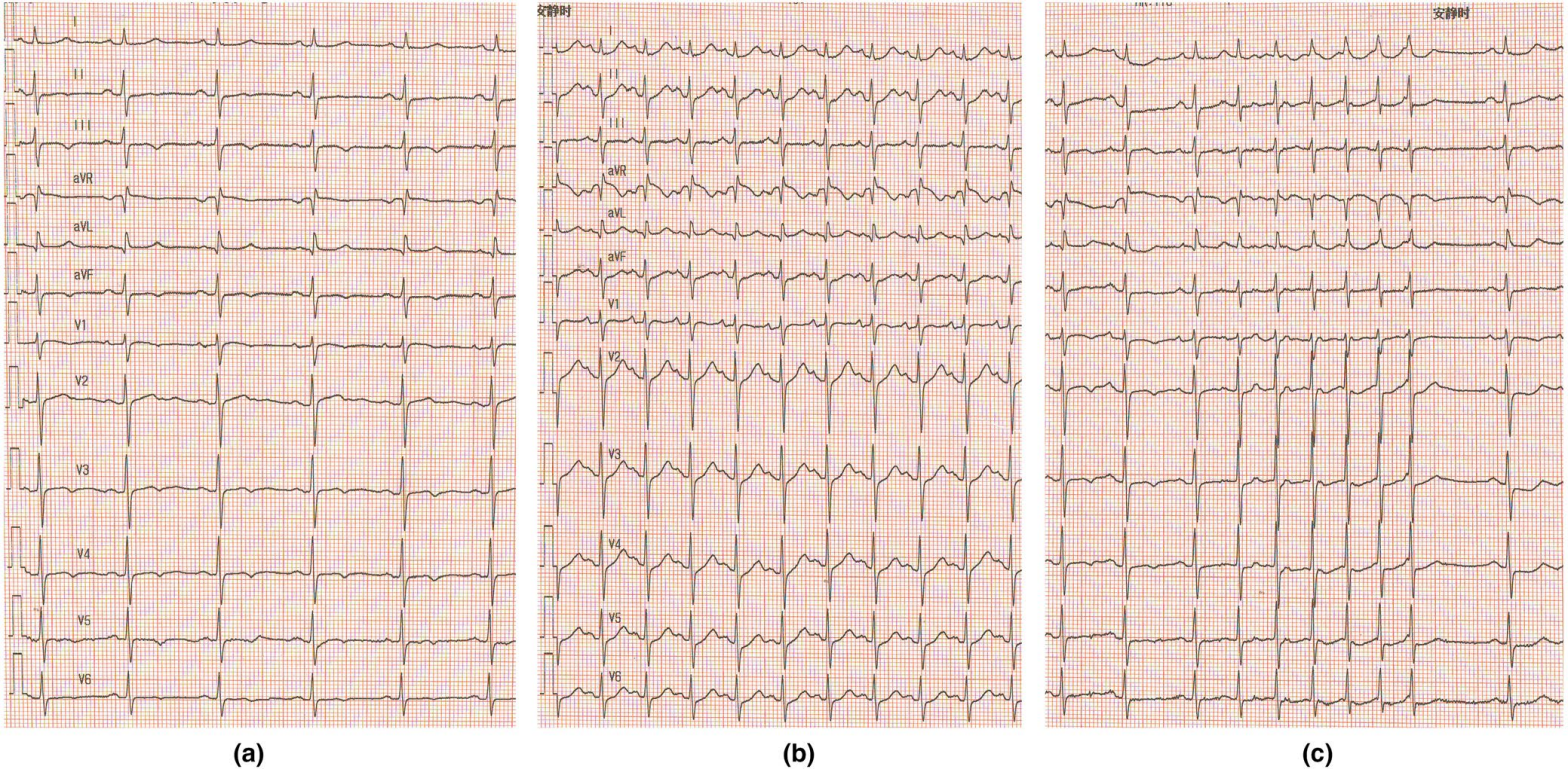
(a) A 12‐lead electrocardiogram (ECG) recording of a sinus rhythm before intravenous isoprenaline administration; (b) A 12‐lead electrocardiogram recording tachycardia detected 1 min after intravenous isoprenaline administration; (c) A 12‐lead electrocardiogram of paroxysmal atrial tachycardia induced by swallowing detected 1 min after intravenous isoprenaline 1 min administration

To clarify the mechanism of tachycardia, we first performed an atropine test. Atropine (1.8 mg for a patient weight of 60 kg) was injected intravenously within 1 min, and the result was negative. Atropine did not inhibit the tachycardia when the patient was swallowing (Figure 2). Next, we administered an intravenous injection of esmolol (0.2 mg kg min^−1^), and the patient's heart rate dropped to 60 bpm, which did not easily induce tachycardia when swallowing (Figure 3). Finally, we administered isoproterenol intravenously, and the heart rate increased significantly. The morphology of the electrocardiogram of the tachycardia during swallowing was consistent with that of tachycardia induced by intravenous infusion of isoproterenol (Figure 4). Therefore, we speculate that the onset of tachycardia during swallowing may have been due to the activation of the sympathetic nerve.

**FIGURE 5:**
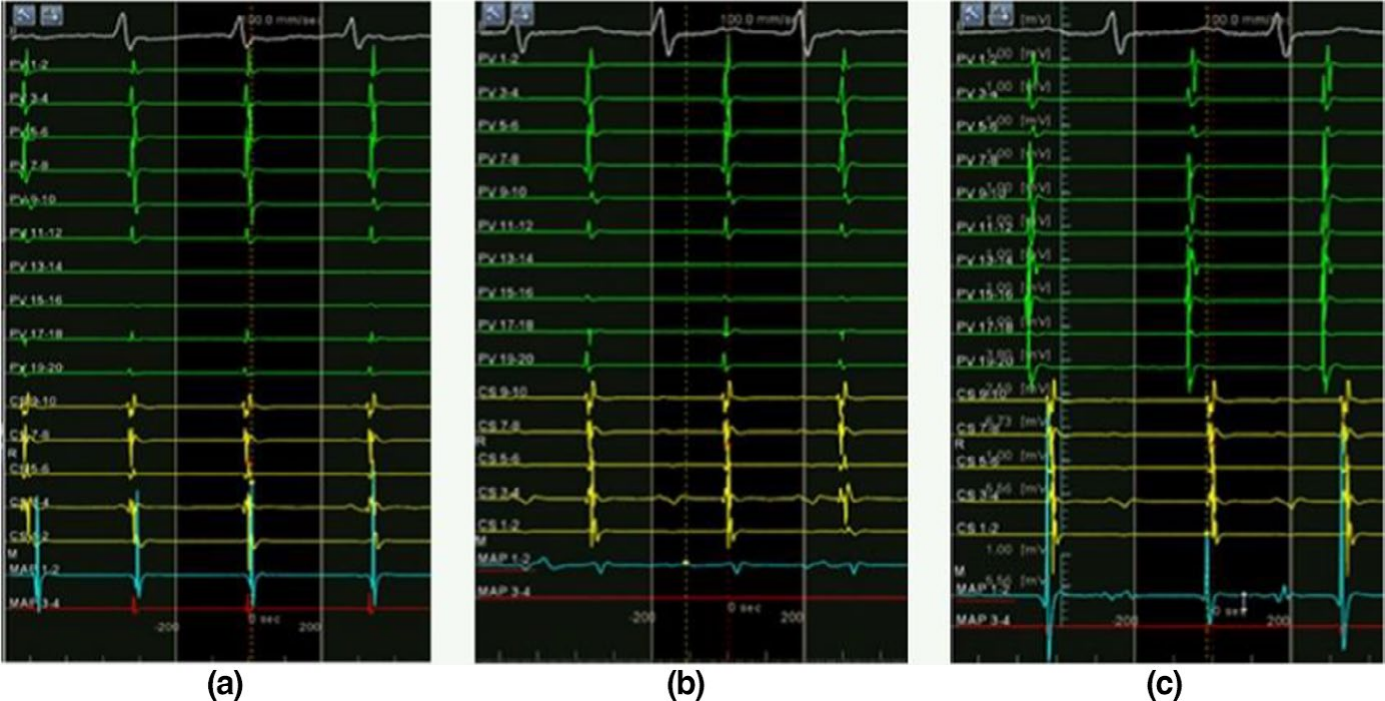
Yellow lines represent potentials of coronary sinus. Blue line represents the potential of the irrigated contact force‐sensing catheter. The potential of the green line is from a PentaRay which is located at the LA‐RSPV junction. (a) Potential of the ablation catheter at the superior vena cava during tachycardia. (b) Potential of the ablation catheter at the coronary sinus ostium during tachycardia. (c) Potential of the ablation catheter at the His bundle during tachycardia

**FIGURE 6:**
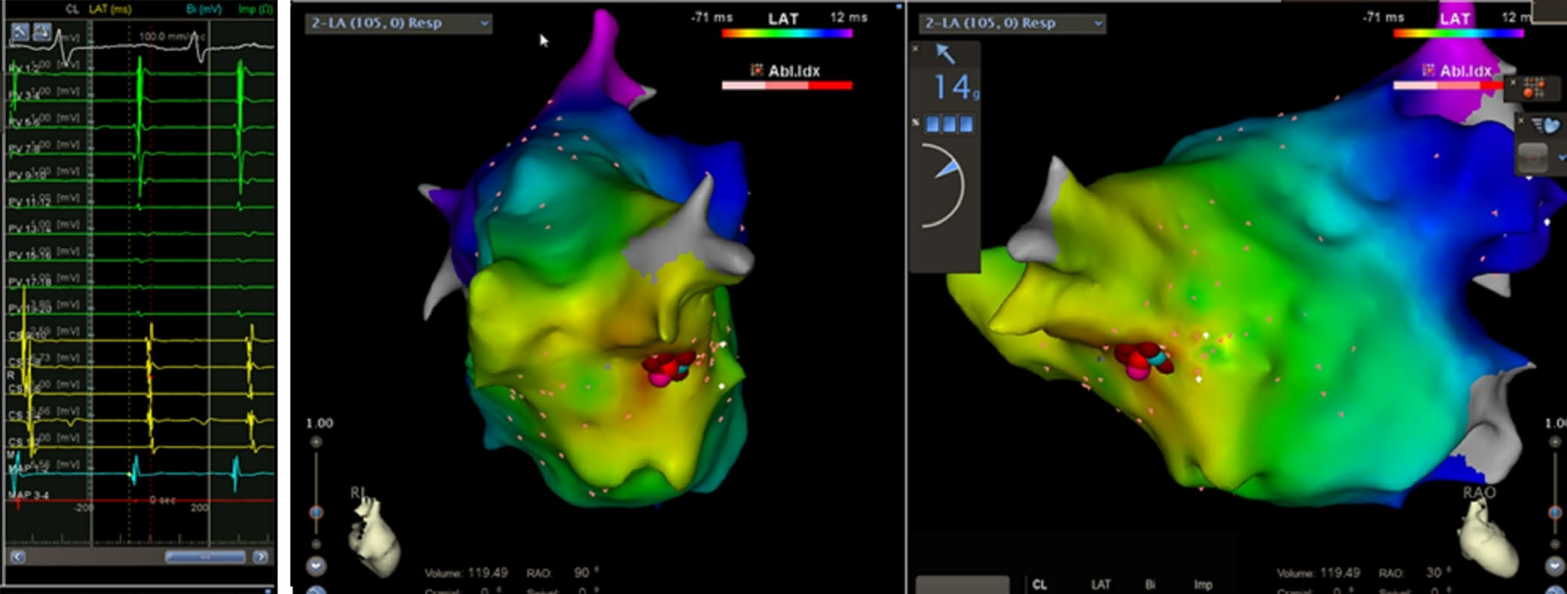
Potential near the LA‐RSPV junction and the ablation target

**FIGURE 7:**
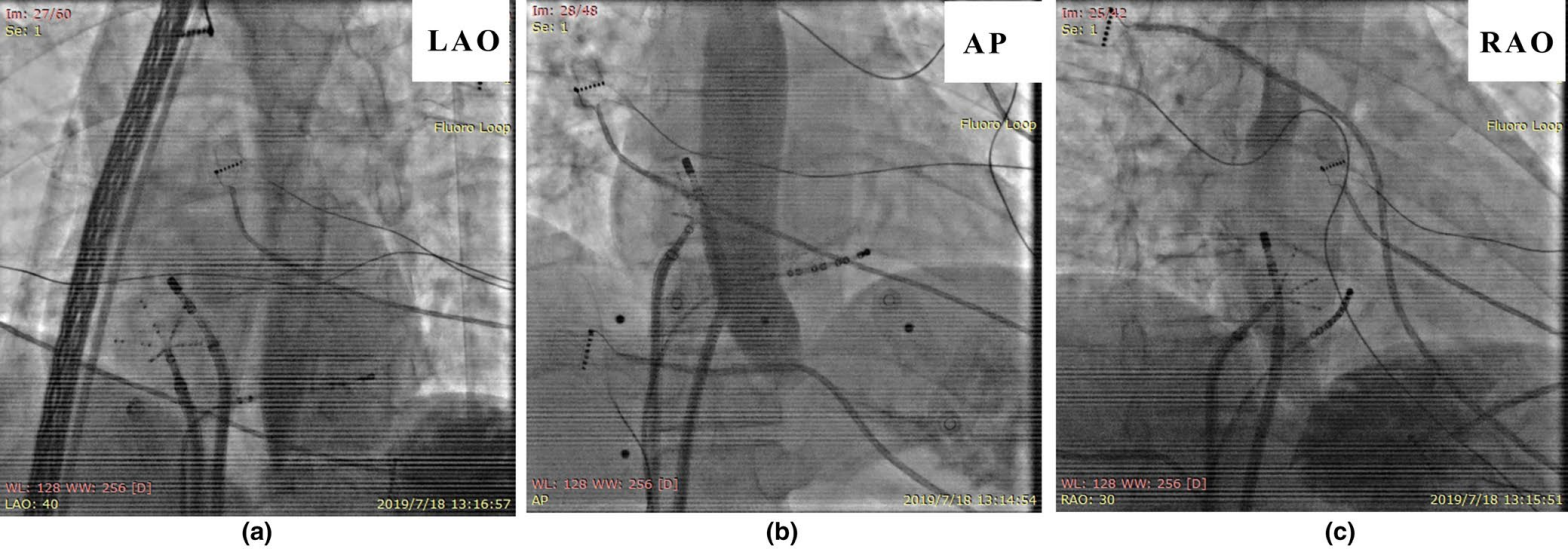
Esophagography revealed that the earliest site of SIAT was not adjacent to the esophagus

**FIGURE 8:**
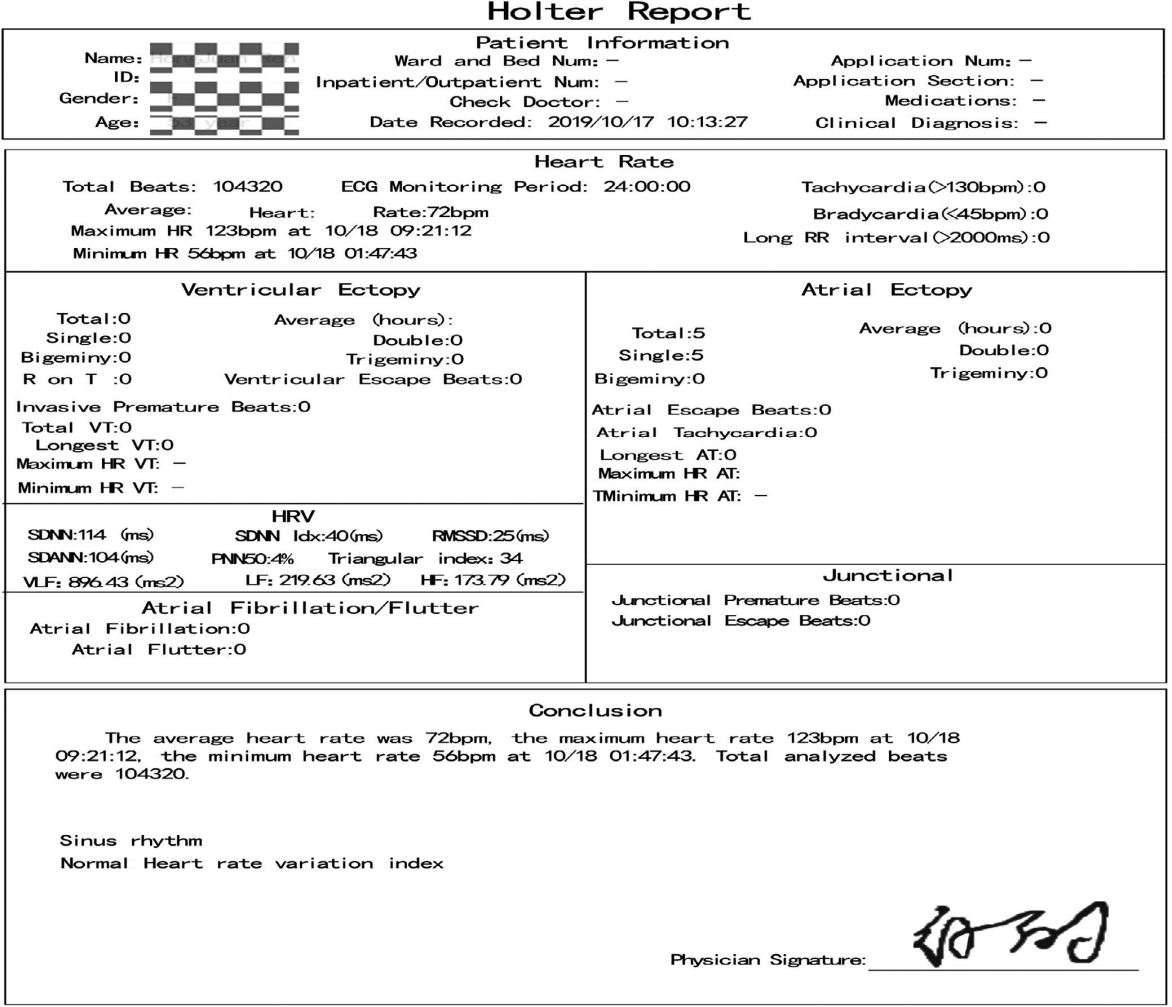
The Holter monitor record 3 months after the radiofrequency ablation

Because palpitations were seriously affect the normal life of the patient, she was strongly recommended to undergo radiofrequency ablation (RFCA). During the procedure, we were able to give her water in a nonsedated state to induce AT. First, an irrigated contact force‐sensing catheter (Navistar Smart Touch, Biosense Webster) was used to create a right atrial activation map using CARTO3 (Biosense Webster) to define the origin of the nonsustained arrhythmia. This indicated that the earliest activated site during induced AT was in the left atrium. For confirmation, we mapped the superior vena cava (SVC), coronary sinus ostium (CSO), and His bundle (Figure 5) but did not find the earliest activated site. Then, we sent a PentaRay catheter (Biosense Webster) into the left atrium and created a left atrial activation map Figure 6, which showed that the earliest activated site was the LA‐RSPV junction. RFCA was performed using an irrigated contact force‐sensing catheter (Navistar Smart Touch, Biosense Webster) with temperature and power limits of 43°C and 35 W, respectively. We did not observe a significant vagus bradycardia response during radiofrequency energy delivery. Finally, tachycardia could not be induced by swallowing after ablation. After the ablation was completed, esophagography was performed to determine the relationship between the ablation target and the esophagus, as shown in Figure 7. Postprocedure, the patient's atrial tachyarrhythmia did not recur, and a 3‐month Holter monitor recording did not show any tachyarrhythmia despite swallowing (Figure 8).

## 3 DISCUSSION

SIAT was first reported by Sakai in 1926 (Tandeter et al., 2010). Although the pathological mechanism of SIAT has not been made clear to date, according to previous case reports, the following mechanisms may be responsible for this rare tachycardia. Firstly, the esophagus is located adjacent to the posterior wall of the left atrium, and mechanical stimulation of the left atrium by a food in the esophagus may induce tachycardia (Bexton, Nathan, Hellestrand, & Camm, 1981). Secondly, SIAT may be a result of a vagal reflex originated by deglutition. Because the vagus nerve is involved in the primary peristalsis of the esophagus, vagal stimulation can make the refractory period of atrial tissue shorter in a nonuniform manner, with repolarization dispersion leading to micro‐reentrant circuits triggered by premature atrial contraction (Alessi, Nusynowitz, Abildskov, & Moe, 1958; Suarez, Chiozza, Foye, Mosso, & Perosio, 1980). Finally, swallowing may activate an adrenergic reflex in the esophagus. Activation of the sympathetic nervous system can lead to nonuniform atrial depolarization and trigger atrial activity via delayed depolarizations. This asynchronous atrial depolarization can lead to focal reentry, resulting in atrial arrhythmias (Kanjwal & Grubb, 2007; Undavia, Sinha, & Mehta, 2006). Most cases reported in the literature are still considered to be associated with vagal activation (Tandeter et al., 2010). The cardiac conduction system is dominated by sympathetic and vagal nerves. The main receptors of the vagus nerve are M receptors, and the main receptors of sympathetic nerves are β1 receptors (Hildreth, Anderson, & Henderson, 2009). Some intracardiac ganglia contain sympathetic, sensory, and peptide transmitters. Esmolol is a selective β1 receptor blocker, and isoproterenol is a β1 receptor agonist that has a strong agonistic effect on β1 and β2 receptors.

In our case, the patient developed tachycardia even with dry swallowing, and during RFCA, esophagography revealed that the earliest site of the SIAT was not adjacent to the esophagus (Figure 7). The above two points can exclude the cause of mechanical stimulation of esophageal dilatation. After we used atropine (an M receptor blocker) in the patient, the tachycardia could still be induced by swallowing, strengthening arguments against a vagal reflex and indicating that a nonacetylcholine neurotransmitter was involved during this process. When we used esmolol, the patient could not induce atrial tachycardia when swallowing, but she could when we used isoprenaline, indicating sympathetic involvement in the induction of the patient's atrial tachycardia. Our ablation target was in the right upper pulmonary vein vestibule, which is near the ganglionated plexus; moreover, we observed no vagal effect at the ablation target site. Therefore, we considered that the ganglionated plexus may contain sympathetic nerves.

There have been previous reports of swallowing‐induced atrial fibrillation, which has led us to consider the prognosis of SIAT. The presence of atrial tachycardia deriving from the pulmonary veins may be misdiagnosed as atrial fibrillation (AF). Since the circumvented pulmonary vein vestibule is the basis of RFCA of AF, these patients were also radically treated. It is also hypothesized that patients with SIAT will develop AF without treatment. For treating SIAT, the following aspects should be considered. First, avoid triggers (e.g., ice‐cold beverages, coffee, or salbutamol) and treat for primary diseases, such as intrapleural repositioning of the esophagus and circular esophageal myotomy. Then, the most commonly used medications were antiarrhythmic drugs. In the last few years, RFCA has become a strategy for treating SIAT. Radiofrequency ablation is a good choice for patients with frequent seizures, poor drug response, and strong RFCA willingness.

## 4 CONCLUSIONS

The case presented here differs from previous reports that presented changes in body position to the left lateral position as a possible causative factor for SIAT. From the above discussion, although it is difficult to illustrate causality in a case report, the conclusion can be reached that a β‐agonist such as isoproterenol may induce tachycardia by activating adrenergic reflexes originating in the esophagus and that a β‐blocker such as esmolol may relieve tachycardia by blocking sympathetic activation while swallowing.

## CONFLICTS OF INTEREST

The authors declare that there are no conflicts of interest regarding the publication of this article.

## AUTHOR CONTRIBUTIONS

All of the authors participated in the conception, design, and writing and revision of the article, and the analysis and interpretation of the data. All of the authors approved the final version of the manuscript to be published and agreed to act as guarantors of the work.

## CONSENT

Informed consent was obtained from this patient for publication of this case history and associated images.

## ETHICAL APPROVAL

The risk of ablation was discussed in detail. The patient gave written informed consent before radiofrequency ablation. The case report was approved by the ethics committee of Beijing Anzhen Hospital affliated with Capital Medical University.
